# Evaluation of Stress Response in Middle-Aged Male Diabetic Hypertensive Patients

**DOI:** 10.1210/clinem/dgad122

**Published:** 2023-03-06

**Authors:** Iliriana Alloqi Tahirbegolli, Bernard Tahirbegolli, Selçuk Şen, Betül Sayın, Mert Kaşkal, Ali Yağız Üresin

**Affiliations:** Clinical Pharmacology Division, Department of Medical Pharmacology, Institute of Medical Sciences, Istanbul University, 34126 Istanbul, Turkey; Laboratory Technician Department, Heimerer College, 10000 Prishtina, Kosovo; Public Health Department, Institute of Medical Sciences, Istanbul University, 34126 Istanbul, Turkey; Management of Health Institutions and Services Department, Heimerer College, 10000 Prishtina, Kosovo; National Sports Medicine Center, 10000 Prishtina, Kosovo; Division of Clinical Pharmacology, Department of Medical Pharmacology, Istanbul Faculty of Medicine, Istanbul University, 34093 Istanbul, Turkey; Division of Clinical Pharmacology, Department of Medical Pharmacology, Istanbul Faculty of Medicine, Istanbul University, 34093 Istanbul, Turkey; Department of Pharmacology, Marmara University School of Medicine, 34854 Istanbul, Turkey; Division of Clinical Pharmacology, Department of Medical Pharmacology, Istanbul Faculty of Medicine, Istanbul University, 34093 Istanbul, Turkey

**Keywords:** hypertension, diabetes mellitus, stress test, salivary cortisol

## Abstract

**Context:**

Stress triggers a cascade of reactions that alter the organism's dynamic steady state. There is a scarcity of interventional studies that show cortisol variability upon stress over time in groups of patients with chronic noncommunicable diseases and comorbidities.

**Objective:**

We aimed to examine salivary cortisol changes in the cognitive stress response of patients with hypertension and diabetes mellitus (HT&DM) and patients with hypertension (HT) and to determine differences between them.

**Methods:**

The study was conducted using a stress test of solving an arithmetic task in 62 patients with HT&DM and HT who were being treated in the outpatient clinic of the Medical Pharmacology and Clinical Pharmacology Department in Istanbul University, Istanbul Medical Faculty Hospital.

**Results:**

There was no statistically significant difference between the HT&DM and HT groups for systolic blood pressure (SBP) and diastolic blood pressure (DBP) values (*P* = .331 and *P* = .058). When measured by repeated ANOVA, salivary cortisol level [F (1.842, 60) = 8.771, *P* < .0001], SBP [F (2.185, 60) = 12.080, *P* < .0001], DBP [F (2.793, 60) = 6.043, *P* = .001], and heart rate [F (2.073, 60) = 13.259, *P* < .0001] were statistically significant for the main effect (time), while the effect of the group × time interaction factor was statistically not significant (*P* = .773; *P* = .751; *P* = .713 and *P* = .506, respectively).

**Conclusion:**

The arithmetic problem-solving task used with the HT&DM and HT patients was useful as an acute stress test in the laboratory environment. There was no statistically significant difference for group × time interaction factor between the HT&DM and HT groups; however, the salivary cortisol and BP values increased significantly after acute stress within each group.

Outside stimuli create stress and stress response in the body at different levels. Stress triggers a cascade of reactions that alter the organism’s dynamic steady state. At the same time, maintaining homeostasis and adapting to stress conditions is a top priority for all organisms. Homeostasis re-establishment and maintenance necessitate neuroendocrine and autonomic nervous system (ANS) control (1). The central nervous system and the endocrine system start the stress response. In particular, the hypothalamus releases corticotropin-releasing factor, and the locus ceruleus releases norepinephrine. The pituitary and adrenal glands are affected by these hormones and come into play (2). It is known that stress affects the quality of life and carries several health risks. While chronic stress raises blood pressure and glucocorticoids, acute stress raises blood pressure, heart rate, plasma glucocorticoid concentration, and blood glucose levels quickly (3).

Massar et al found that patients with less sleeping time have higher blood pressure and cortisol release (4). Young adults who respond to psychological stress with higher blood pressure reactivity are at higher risk for developing hypertension when they reach middle age (5). Additionally, Hamer and Steptoe found in their research that those among healthy subjects who responded with higher cortisol reactivity after psychological stress testing had a higher risk of developing hypertension later (6). Salivary cortisol and α-amylase may be useful diagnostic tools for cardiovascular disease, especially in explaining the risk of cardiovascular disease caused by stress (7). It is known that stress may also lead to glycemic control disorder and lifestyle changes in diabetic individuals. Furthermore, it is known that hypothalamic-pituitary-adrenal activity in type 2 diabetic mellitus (T2DM) patients is higher in those with diabetes complications and that diabetic complications are associated with cortisol release (8). In addition, Hamed et al found that hypercortisolemia could worsen cognitive dysfunction in T2DM patients with poor plasma glucose control in their study (9). Salzmann et al found that short psychological support to reduce pretest stress response is effective in improving salivary cortisol response in healthy subjects (10).

Early studies focused on the response to stress in children, young and healthy adults, and patients with T2DM, specifically the impact on blood pressure (BP), heart rate, blood glucose level, sleep, and other factors (3-6, 10, 11). However, there is a scarcity of interventional studies that show cortisol variability on stress over time in groups of patients with chronic noncommunicable diseases and comorbidities. Furthermore, we consider that there is a dearth of comparative research examining the response to stress in male hypertensive patients vs patients with hypertension and diabetes mellitus. In this research, we aimed to examine salivary cortisol changes in the cognitive stress response of male patients with hypertension and diabetes mellitus (HT&DM) and hypertension (HT) and to determine the differences between them.

## Methods

This clinical study was carried out during March to December 2017 period with a total of 62 outpatients diagnosed with HT&DM and HT at Istanbul Medical Faculty, Department of Clinical Pharmacology Polyclinic. The inclusion criteria were male sex; being on antihypertensive treatment for the last 3 months; diagnosed for T2DM with antidiabetic drug use for at least 3 months; and being between 40 and 65 years of age. Exclusion criteria were female gender; patients under the age of 40 or over the age of 65; uncontrolled blood pressure (systolic BP [SBP] > 140 mmHg, diastolic BP [DBP] > 90 mmHg); antipsychotic medication use; being on current treatment for less than 3 months; having a previous myocardial infarction; having a previous stroke, lung disease, organ failure, Cushing disease, or aldosteronism; and failing to sign the informed consent form. Patients who could not complete the stress test, provide a total of 4 saliva samples during the study, or had too high a blood pressure reaction to stress (SBP > 190 mmHg and DBP > 100 mmHg) were excluded from the study or had their data collection stopped. The G*Power 3.1 program calculated 0.25 effect size and 0.05, 1- were 0.99 for 4 repeated measurements in the post hoc sample calculation on the repeated measures ANOVA axis for our sample.

Saliva samples were collected in our study for cortisol measurement, and patients were asked to insert the cotton piece in the salivette tubes directly into their mouths without touching it with anything, hold it for 30 to 60 seconds, and then insert it back into the tubes in the same way. The electrochemiluminescence immunoassay method, with Roche Elecsys 2010 Immunoassay Analyzer (RRID:SCR_020499), was used to measure salivary cortisol.

Saliva cortisol concentration is strongly correlated with serum cortisol level, and changes in free cortisol concentration are reflected in salivary cortisol concentration within a few minutes (12). Hellhammer et al reported that salivary cortisol is a useful biomarker for assessing stress response in individuals with chronic diseases who volunteered to participate in research (13).

In this study, to induce psychological stress in the laboratory setting and to influence poststress cortisol reactivity, the stress test used was to solve an arithmetic problem, specifically, the modified form of the arithmetic task used by Uchino et al (14).

In a quiet setting, the patients were asked to subtract 1 or 2 numbers within 10 minutes from 6 different 4-digit numbers, as a mathematical problem-solving task. A4 white paper was placed in front of the patient, and they were instructed to use the subtraction function to calculate each given number within 100 seconds. The researcher was instructed to stand in front of the patient and make corrections by issuing a warning whenever the patient made a calculation error.

After the first 100 seconds, the second set of numbers was given and the participant was asked to continue, and so forth. For the 10-minute test, the following numbers must be calculated: From 0 to 100 seconds: 2907 − 3; 101 to 200 seconds: 6828 − 7; 201 to 300 seconds: 9561 − 13; 301 to 400 seconds: 5113 − 8; 401 to 500 seconds: 8318 − 14; and 501 to 600 seconds: 9994 − 17.

**Figure 1.**
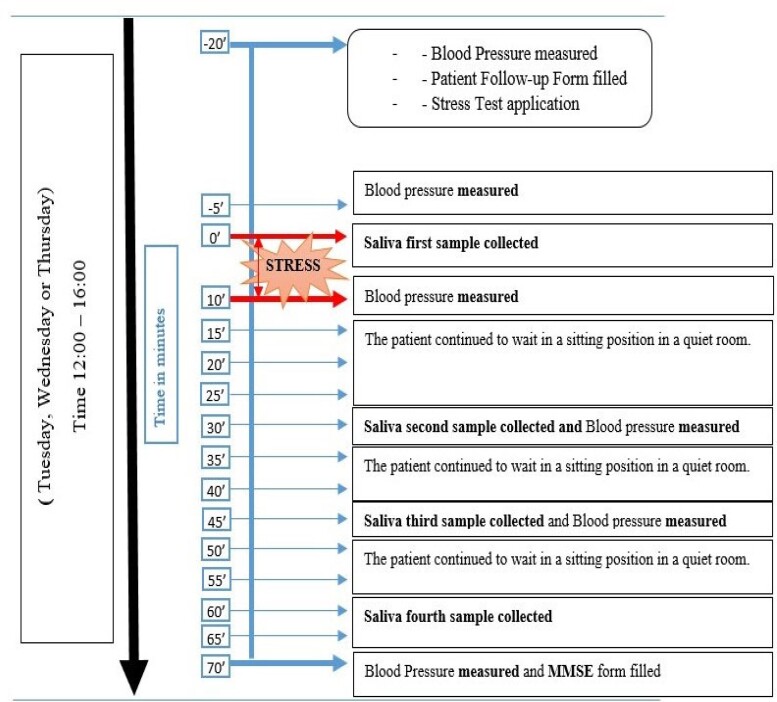
The study protocol.

We conducted the stress test on Tuesdays, Wednesdays, and Thursdays to avoid additional stress stimuli at the beginning of the week and at the weekend. When the patient arrived in the afternoon for the test, the first blood pressure measurement and pulse per minute count were taken (12:00 to 16:00). After that, the patient sat for 15 minutes to rest. Salivary samples were taken with salivette tubes 4 times in total, 1 time before arithmetic stress and 3 times after stress (Fig. 1).

After the volunteers arrived and rested for at least 5 minutes, a physician-researcher measured systolic and diastolic BP and heart rate per minute using an electronic blood pressure measurement device (OMRON 705IT model) on the right arm at heart level in a sitting position.

Following the completion of the test, the patient completed the Mini-Mental State Examination (MMSE) form with the assistance of the researcher in order to evaluate cognitive function (15). The MMSE Turkish validity and reliability research by Gürgen et al in 2002 showed acceptable reliability (16).

In the study, the sociodemographic information of the patients (year of birth, marital status, education, occupation, family structure, economic status, and how many years they had diabetes and hypertension) was asked and recorded by the researcher. In the second part of the information form, the drugs used by the patients were recorded.

The continuous variables are summarized by mean and SD or median (med) and interquartile range (IQR); qualitative data are summarized as frequency (n) and percentage (%). The normal distribution of continuous variables was examined by Kolmogorov-Smirnov and Shapiro-Wilk tests. Differences between variables were assessed using the independent groups *t* test or Mann Whitney U test, Chi-square or GLM repeated measures test. Analysis of the data was done using the Windows SPSS v21.0 package program and *P* < .05 was considered statistically significant. This study's procedures were fully compliant with the provisions of the Helsinki Declaration on Human Subjects Research. All the enrolled patients gave their written informed consent and the study's protocol and data collection tools were approved by the Istanbul University Istanbul Medical Faculty Ethics Committee on June 24, 2016 (No: 901-2016/829). This study was registered at ClinicalTrials.gov (NCT05662930).

## Results

The mean age of the participants was 53 ± 6 years for the HT group and 55 ± 6 years of age for the HT&DM group. The majority of participants were married and had equal income to outcome economic status. There was no statistically significant difference among HT&DM and HT groups on age (*P* = .361), body mass index (*P* = .403), family structure (*P* = .698), education (*P* = .330), SBP (*P* = .331) or DBP (*P* = .058), and heart rate values (*P* = .666) (Table 1). There was no statistically significant difference among HT&DM (median [IQR]: 235.0 [171.7–350.0]) and HT (252.0 [188.5–322.2]) on the baseline level of salivary cortisol (*P* = .868) (Table 1).

**Table 1.** Sociodemographic data for participant groups

	HT&DM group (n = 20) mean ± SD or median (IQR) or n (%)	HT group (n = 42) mean ± SD or median (IQR) or n (%)		*P*
Age, years	55.45 ± 6.32	53.79 ± 6.80	t = 0.921	.361
BMI	29.49 ± 5.46	28.41 ± 4.25	t = 0.842	.403
Marital status				
Married	18 (90.0)	40 (95.2)	*x* ^2^ = 3.351	.341
Single	1 (5.0)	0 (0.0)		
Divorced	1 (5.0)	1 (2.4)		
Widowed	0 (0.0)	1 (2.4)		
Family status				
Nuclear	16 (80.0)	34 (81.0)	*x* ^2^ = 0.719	.698
Extended	1 (5.0)	4 (9.5)		
Single-parent	3 (15.0)	4 (9.5)		
Education				
Illiterate	0 (0.0)	1 (2.4)	*x* ^2^ = 4.605	.330
Primary school	4 (20.0)	15 (35.7)		
High school	6 (30.0)	15 (35.7)		
University	6 (30.0)	8 (19.0)		
Master/Doctorate	4 (20.0)	3 (71.0)		
Economic status				
Outgoings more than income	6 (30.0)	9 (21.4)	*x* ^2^ = 1.614	.446
Income equal to outgoings	10 (50.0)	28 (66.7)		
Outgoings more than income	4 (20.0)	5 (11.9)		
Time from diabetes diagnosis, years	6 (3–10)	NA	NA	NA
Time from hypertension diagnosis, years	6 (2–10)	5 (2–9)	U = 393.5	.949
Systolic blood pressure, mmHg	129 ± 10	132 ± 9	t = 0.980	.331
Diastolic blood pressure, mmHg	76 ± 8	81 ± 10	t = 1.929	.058
Heart rate	76 ± 9	77 ± 11	t = 0.434	.666
MMSE	27.37 ± 2.16	26.83 ± 2.19	t = 0.888	.378

Abbreviations: BMI, body mass index (kg/m^2^); DM, diabetes mellitus; HT, hypertension; MMSE, Mini-Mental State Examination.

Analyzed with repeated measures ANOVA, salivary cortisol level (F [1.842, 60] = 8.771, *P* < .0001), SBP (F [2.185, 60] = 12.080, *P* < .0001), DBP (F [2.793, 60] = 6.043, *P* = .001) and the heart rate (F [2.073, 60] = 13.259, *P* < .0001) were statistically significant for the main effect (time), while the effect of the group × time interaction factor was statistically not significant (*P* = .773; *P* = .751; *P* = .713; and *P* = .506, respectively) (Table 2).

**Table 2.** Assessment of salivary cortisol values with ANOVA test for both research groups

	Time			Group			Time × Group		
	*F*	d.f.	*P*	*F*	d.f.	*P*	*F*	d.f.	*P*
Salivary cortisol	8.771	1.842, 60	<.0001	0.697	1, 60	.407	0.235	1.842, 60	.773
Systolic blood pressure	12.080	2.185, 60	<.0001	2.590	1, 60	.113	0.313	2.185, 60	.751
Diastolic blood pressure	6.043	2.793, 60	.001	3.930	1, 60	.052	0.437	2.793, 60	.713
Heart rate	13.259	2.033, 60	<.0001	0.125	1, 60	.725	0.702	2.033, 60	.506

**Figure 2.**
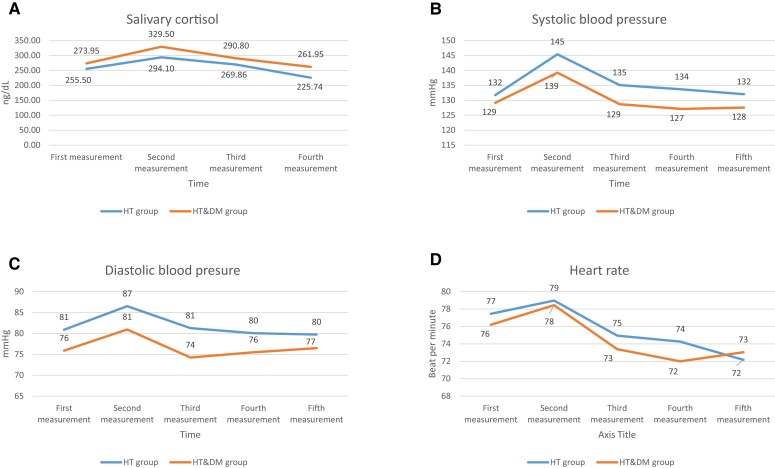
Changes in salivary cortisol values (A), systolic blood pressure (B), diastolic blood pressure (C), and heart rate (D) during the stress test and after the test for both groups.

In the HT&DM group, there was a statistically significant difference between the first (mean ± SD: 273.9 ± 131.1) (ng/dL) (before the stress test) and second measurements of salivary cortisol values (20 minutes after stress test) (*P* = .017), while the third (290.8 ± 154.5) (35 minutes after stress test) and fourth measurements (261.9 ± 145.5) (50 minutes after stress test) were statistically significantly lower than the second measurement (329.5 ± 197.8) (*P* = .007 and *P* = .001). There was no statistically significant difference between the first (255.5 ± 91.6) (before the stress test) and second (294.1 ± 127.5) (20 minutes after stress test) measurements of salivary cortisol values in the HT group (*P* = .052), while the fourth (225.7 ± 124.9) (50 minutes after stress test) measurements were found to be statistically significantly lower than the second (294.1 ± 127.5) (20 minutes after stress test) (*P* < .0001), and third (269.9 ± 127.5) (35 minutes after stress test) measurements (*P* < .0001) (Fig. 2A).

The second measurement of SBP (139 ± 12) (mmHg) in the HT&DM group showed a statistically significant increase compared to the first measurement (129 ± 10) (before the stress test) (*P* = .001), and the third (129 ± 13) (*P* < .0001), fourth (127 ± 16) (*P* < .0001), and fifth (127 ± 16) (*P* = .002). Systolic blood pressure values in the HT group increased statistically significantly in the second measurement (145 ± 16) (mmHg) compared with the first measurement (132 ± 9) (*P* < .0001) (before the stress test). The third (135 ± 14), fourth (134 ± 15), and fifth measurements (134 ± 15) were found to be statistically significantly lower than the second measurements (145 ± 16) (*P* < .0001; *P* < .0001; and *P* = .002; respectively).

The second measurement (86 ± 13) (mmHg) of DBP values in the HT group showed a statistically significant increase compared with the first measurement (81 ± 10) (before the stress test) (*P* = .002), and the third (81 ± 11), fourth (80 ± 12), and fifth (80 ± 17) measurements were found to have a statistically significant decrease compared with the second measurement (86 ± 13) (*P* = .003, *P* < .0001, and *P* = .008, respectively). There was no statistically significant difference between DBP measurements in the HT&DM group before (76 ± 8) and after the stress (second: 81 ± 10; third: 74 ± 10; fourth: 75 ± 9; and fifth: 76 ± 7 mmHg, measurements) (consecutively: *P* = .075, *P* = .349, *P* = .742, and *P* = .664). The third diastolic blood pressure measurement was statistically significant lower compared to second measurement (*P* = .030).

While there was no statistically significant difference between the first (77 ± 11) (before the stress test) and second (79 ± 12) heart rate count measurements in the HT group (*P* = .138), the third (75 ± 10), fourth (74 ± 10), and fifth (72 ± 15) measurements were found to be statistically significantly lower than the second measurement (79 ± 12) (*P* < .0001). There was no statistically significant difference between the first (76 ± 9) (HR/min) and second (78 ± 10) measurements in the HT&DM group (*P* = .148) and a statistically significant difference was found between the third (73 ± 8), fourth (72 ± 7) and fifth (73 ± 8) measurements compared with the second (78 ± 10) measurement (*P* < .0001). Figure 2A-2D shows the values of salivary cortisol, systolic and diastolic BP, and heart rate according to the time of the measurements for the HT and HT&DM groups (Fig. 2A-2D).

When using the *t* test to compare the cognitive function, there was no statistically significant difference between the 2 groups (HT and HT&DM) on the MMSE test scores (*P* = .378), even when analyzing with chi-square test, and the scores were divided as dichotomous, where 27 and above was defined as normal and 20 to 26 points as mild regression (*x*^2^ = 1.138, *P* = .286).

**Figure 3.**
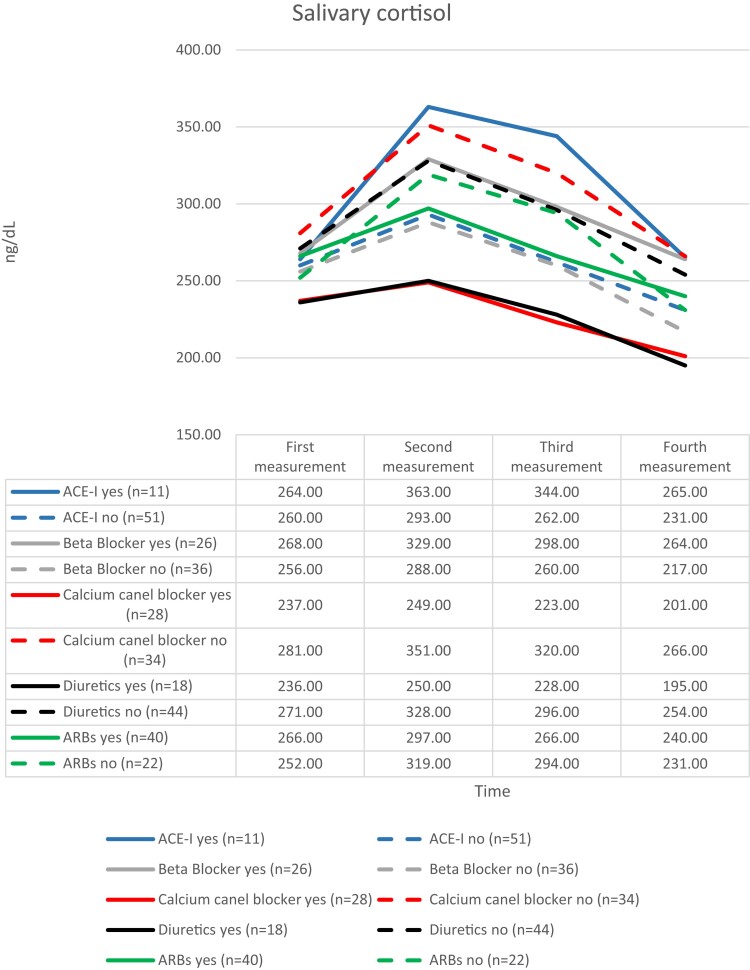
The salivary cortisol values evaluated separately according to the antihypertensive drug groups that participants used or did not use. Abbreviations: ACE-I, angiotensin-converting enzyme inhibitor; ARB, angiotensin receptor blockers.

The variability of cortisol values before and after stress was seen to be smaller among patients who were using antihypertensive therapy with calcium channel blockers and diuretics (Fig. 3).

## Discussion

In this research, we examined the salivary cortisol changes in the cognitive stress response of male patients with hypertension and diabetes mellitus (HT&DM) and hypertension (HT) and the differences between them. The main finding of our study indicates no statistically significant difference in salivary cortisol levels between the HT&DM and HT groups in terms of the group × time interaction factor. However, salivary cortisol and BP values significantly increased within each group following the acute stress test task, which was an arithmetic problem-solving task.

We measured the reactivity of salivary cortisol in this study by taking 4 samples from the onset of stress to 60 minutes, as done in many other studies. Wirtz et al, for example, completed 8 samples in 80 minutes, Hamer and Steptoe completed 5 samples in 75 minutes, Schmidt-Reinwarld et al completed 5 samples in 100 minutes, and Wüst et al collected 4 samples within 60 minutes (6, 17-19).

Salivary cortisol responses to acute stress are known to be significantly influenced by factors such as age and gender, endogenous and exogenous sex steroid levels, pregnancy, lactation, breastfeeding, smoking, coffee, and alcohol intake, as well as dietary energy supply (20).

Furthermore, to avoid being influenced by cortisol's daily biological values, measurements were taken in the afternoon hours, when cortisol is less active. Balodis et al also preferred afternoon hours to perform the test in their study, which is similar to our approach (21).

In this study, although the salivary cortisol values of HT&DM group patients before the stress showed a lower tendency, no statistically significant difference was found between the 2 groups (HT&DM and HT).

Reynolds et al found that high plasma cortisol levels in DM patients were associated with high fasting plasma glucose (22). In another study, plasma cortisol levels were found to be higher in those with glucose intolerance than in healthy subjects (23). Gluck et al found that morning cortisol levels were higher in women with binge eating disorder than in women without binge eating disorders (24). In their studies, Faulenbach et al discovered that cortisol reactivity after the postprandial period stress test in T2DM patients was lower than after the test on an empty stomach (25).

There was no statistically significant difference also in blood pressure (SBP and DBP) between the 2 groups when evaluated in terms of the group × time interaction factor. Inability to detect blood pressure differences between the 2 groups is considered to be related to the inclusion criteria used in the study, since it is desirable that the included subjects be controlled in terms of blood pressure (SBP < 140 mmHg, DBP < 90 mmHg).

However, Wirtz et al in their study found that hypertensive subjects responded with higher SBP and DBP after stress than normotensive subjects (19). Uchino et al also found a statistically significant increase in SBP and DBP and heart rate in young women after acute stress (14).

Another finding of our study was that the use of antihypertensive drug groups was found to be associated with different levels of salivary cortisol activity after stress. Patients who used angiotensin-converting enzyme (ACE) inhibitors had higher reactivity, whereas those who used diuretics and calcium channel blockers had lower cortisol variation in saliva after stress (Fig. 3).

In this research, we found no statistically significant difference between the first (baseline) measurement before the stress test and the second measurement of salivary cortisol values 20 minutes after the stress test in either group, HT&DM or HT. Wirtz et al found in their study that those who had HT had a higher cortisol release activity after stress than those who were normotensive (19). Epel et al found that healthy subjects that had higher cortisol reactivity after stress testing had eaten more caloric food than those who had lower cortisol reactivity (26). However, in our study, higher salivary cortisol levels in those with HT&DM were thought to be associated with higher plasma glucose levels in this group. Furthermore, the control of BP and the poststress salivary cortisol activity in both groups was thought to be affected, due to the stress test performed on a full stomach and in the afternoon, and the absence of known diabetes complications. It is well known that hypertension and cognitive function regression are common conditions in the elderly. It is stated in the Kodl and Seaquist reviews that there is a decline in cognitive function in those with T2DM, and it is also known that glucose control has an effect (27). Better diabetes control and fewer diabetes complications were associated with less cognitive dysfunction (27).

To fully interpret the salivary cortisol values of the subjects in the study, more measurements during the day evaluating the daily cortisol circadian rhythm are needed (28). During the study, it was found that the subjects participated in the study in a variety of psychological states. However, because this was not considered a primary finding and was intended to be used as an exclusion criterion only if the patients used any substance from the psychoactive drug group, the psychological mood scale was not used. It may be an important finding in future studies to evaluate the volunteers’ depression and anxiety levels at the time of their participation in the study.

In conclusion, the arithmetic problem-solving task used in the HT&DM and HT patients were found to be useful as an acute stress test in the laboratory environment. In the HT&DM and HT groups, salivary cortisol and BP values increased significantly after acute stress, but no statistically significant difference was found in terms of group × time interaction factor. It was discovered that various antihypertensive drug groups are associated with different levels of salivary cortisol activity after stress. Despite this, little is known about the effect of these antihypertensives on cortisol levels after acute stress, and research on the effect of different antihypertensives on stress response modification is lacking.

## Data Availability

The data that support the findings of this study are available from the corresponding author upon reasonable request.

## Clinical Trial Registration

This study was registered at ClinicalTrials.gov (NCT05662930).

## Abbreviations

BP: blood pressure
DBP: diastolic blood pressure
HT: hypertension
HT&DM: hypertension and diabetes mellitus
IQR: interquartile range
MMSE: Mini-Mental State Examination
SBP: systolic blood pressure
T2DM: type 2 diabetes mellitus
